# Prevalence of chronic respiratory diseases diagnosed by pulmonary function testing: a cross-sectional study

**DOI:** 10.1097/MS9.0000000000002884

**Published:** 2024-12-19

**Authors:** Sarah Abualgasim Musa Alnoor, Omer Elgaili Yousif Elhag, Najlaa Mohammed Abass Ali, Yousif Omer Elgaili Yousif, Asmaa Abdelmaged Eltaieb Mohamed, Awab Hashim Sulieman Saad, Amro Abdalmageed Altayeb Mohamed

**Affiliations:** a Respiratory Medicine Department, Sudan Medical Specialization Board, Khartoum, Sudan; b Respiratory Medicine Department, Faculty of Medicine, Alneelain University, Khartoum, Sudan; c Faculty of Medicine, University of Bahri, Khartoum Sudan; d Surgery Department, Alzaiem Alazhari University, Khartoum, Sudan; e University of Medical Sciences and Technology, Khartoum, Sudan

**Keywords:** chronic respiratory diseases, obstructive respiratory diseases, pulmonary function test, restrictive respiratory diseases

## Abstract

**Background:**

Respiratory diseases are pathological conditions affecting the organs and tissues involved in gas exchange. Pulmonary function tests allow for the classification of the severity of respiratory diseases, their follow-up, and treatment response assessment. The aim was to determine the prevalence of chronic respiratory disease and the risk factors in Khartoum, Sudan.

**Methods:**

A descriptive cross-sectional hospital-based study was conducted in the Respiratory Department from May to October 2021, including the patients who underwent pulmonary function tests. Data were analyzed using Statistical Package for Social Sciences, version 25.0.

**Results:**

Out of the 396 study participants, 214 (54%) were above 40 years of age with similar gender distribution and a male-to-female ratio of 1.1: 1, and almost all were Sudanese. Clinically, 40.4% had normal body mass index (BMI), 24.2% underweight, and 13.6% were obese. Smokers represented 19.4% of the study participants. Moreover, 18.2% reported a positive history of bronchial asthma. Regarding pulmonary function test patterns, 50.8% exhibited normal results, 25.3% displayed restrictive patterns, 19.7% showed obstructive patterns, and 4.3% had mixed patterns. Chi-square cross-tabulation testing revealed a significant association between older ages of participants and the abnormal pulmonary function test results (*P* < 0.001).

**Conclusion:**

The study revealed that the proportion of respiratory diseases with abnormal lung function test results is considerable and should not be ignored, especially among older patients. Therefore, when indicated, attention should be paid to performing lung function tests widely to accurately identify the prevalence rates of lung diseases and associated risk factors in Sudan.

## Introduction

Chronic respiratory diseases (CRDs) are a collection of illnesses affecting the airways that work together to gradually deteriorate lung function. Alpha-1 antitrypsin deficiency, idiopathic pulmonary fibrosis, asthma, and chronic obstructive pulmonary disease (COPD) are among them. A substantial part of the pathophysiology of these disorders is attributed to dysregulated inflammatory processes^[1]^.

CRDs are responsible for a high burden of morbidity – especially from poorly controlled asthma – and mortality – especially from COPD – causing around 4 million deaths every year globally^[2]^. Post-tuberculosis lung disease is also increasingly recognized as an essential cause of chronic lung disease (CLD), especially in high tuberculosis burden settings^[3]^. An estimated 500 million people were living with CLD in 2017, an increase of 40 % compared to 1990^[4]^.

The prevalence of COPD is expected to increase as populations continue to age, especially in low- and middle-income countries where 90 % of the global COPD mortality currently occurs, and the population is exposed to biomass use, smoking, and occupational risk factors^[5]^. Though data are sparse, a systematic review of nine cross-sectional studies from sub-Saharan Africa (SSA) has revealed that the prevalence of COPD ranges from 4.1% to 24.8 %^[6]^. A variety of lung illnesses known as pneumoconiosis are brought on by breathing in mineral dust, typically as a result of specific jobs. Chronic pulmonary inflammation and progressive pulmonary fibrosis are the primary pathological characteristics, which might ultimately result in mortality from heart and/or respiratory failure. Globally, pneumoconiosis is common and poses a major risk to public health. Inadequate occupational protection, a dearth of early detection techniques, and ineffective therapies are the main causes of its high prevalence and fatality^[7]^. This variation in prevalence depicts the need for further studies in the understudied populations.

Investigations in respiratory diseases are broadly classified as tests that aid with diagnosis; tests that assess disease severity; and tests that assess disease activity, those tests are non-invasive biomarkers, enabling serial measurement and may inform therapy. One of the challenges of respiratory medicine is the limited spectrum of clinical expression associated with a diverse spectrum of pathologies. Clinical symptoms in respiratory medicine are often of poor specificity for securing a diagnosis or assessing disease severity. Thorough investigations, therefore, necessarily form a critical part of assessment. The most appropriate choice of investigation is an essential component of clinical decision-making that affects patient care and may be influenced by several history questions that the clinician may consider. Due to the lack of such a study in Sudan, we have conducted this study to determine the prevalence of CRDs and the associated risk factors.

## Materials and methods

### Study design and settings

A descriptive cross-sectional hospital-based study was conducted in a governmental hospital. It is located in the center of Khartoum city, Sudan. This hospital is considered an essential center in providing health care for respiratory system disease patients and other relevant health services for the general population from surrounding residential areas and for referred patients from all over the country between May 2021 and October 2021. The study population was all patients with pulmonary function tests at the hospital. The methodology in this study has been reported according to Strengthening The Reporting Of Cohort Studies in Surgery guidelines 2021^[8]^.

### Inclusion criteria

All patients who underwent pulmonary function tests at Alshaab Hospital between May 2021 and October 2021.

- Patients who underwent pulmonary function testing for respiratory symptom evaluation.
- Patients with a confirmed diagnosis of CRDs based on pulmonary function test results.
- Adults above 18 years of age and with signed informed consent.

### Exclusion criteria

Refusal of participation in the study.

- Patients without documented pulmonary function test results.
- Patients with incomplete medical records or missing data for analysis.
- Individuals with acute respiratory conditions without a history of CRDs.

### Sampling

The total coverage method was used to recruit participants; all participants who met the inclusion criteria were involved in the study.

### Data collection methods and tools

Data were collected using a record of pulmonary function tests using a hand-held spirometer (Spiro sound). The test was repeated three times to ensure a reliable result.

### Data analysis

Data were reviewed, ordered, and coded, and then the appropriate statistical tests were used to study the frequency of CRDs. Statistical analysis was performed using the Statistical Package for Social Sciences, version 25.0. We analyzed and described data using R software version 4.0.2. Continuous data were presented as mean ± SD, and categorical data were presented as numbers (percentage). We used the Kolmogorov–Smirnov test to check the normality of the data. To find a significant difference between groups, we used an independent *t*-test for normally distributed data and Mann–Whitney *U* after rejecting the null hypothesis of the Kolmogorov–Smirnov test of normal distribution. We used the Chi-square test or Fisher exact test to find if there was a significant difference between the groups for categorical data. A *P* value less than 0.05 is considered significant.

### Ethical considerations

The Sudan Medical Specialization Board ethical committee approved the proposal for ethical clearance. The serial number assured the confidentiality of the obtained results. Written Consent was considered.

## Results

**Figure 1.**
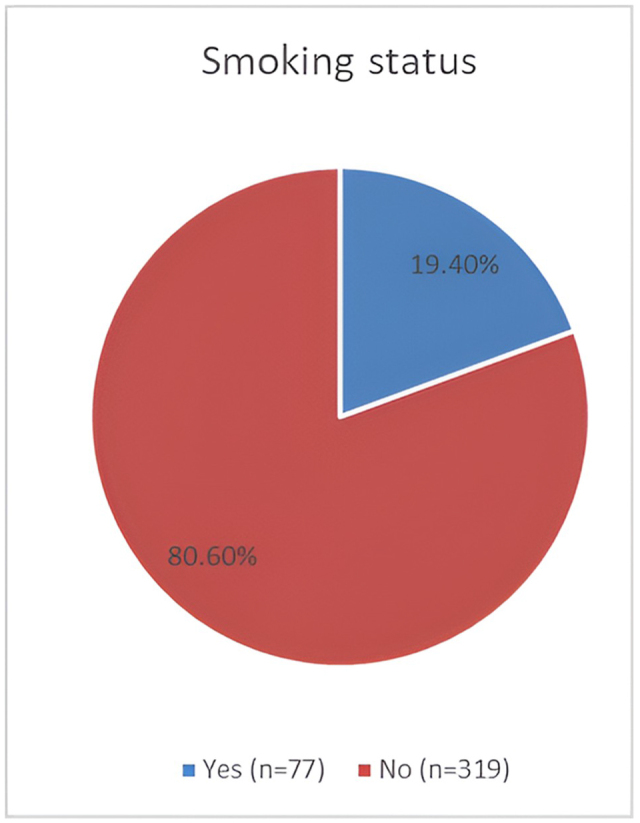
Smoking status in study participants.

**Figure 2.**
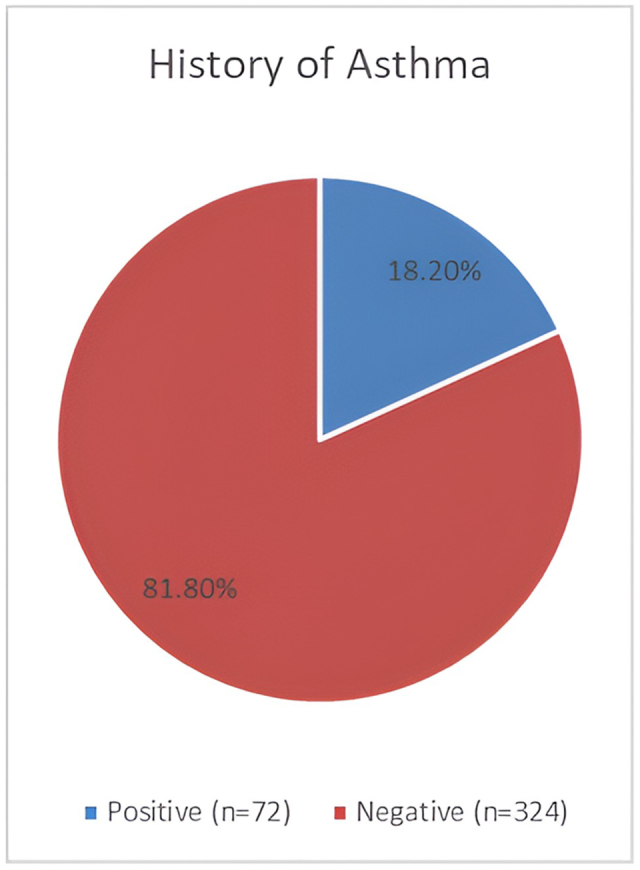
History of asthma among participants.

Three hundred ninety-six participants were enrolled in our study. More than half of them, 214 (54%), were above 40 years of age, with a similar gender distribution and male-to-female ratio of 1.1: 1. Almost all of them were Sudanese, as detailed in Table 1. Clinically, nearly half of the participants, 160 (40.4%), reported normal BMI, 96 (24.2%) were underweight, and 54 (13.6%) were obese, as detailed in Table 2. As shown in Fig. 1, smokers represented 77 (19.4%) of the participants in our study. Moreover, 73 (18.2%) of them reported a positive history of bronchial asthma, as shown in Fig. 2. The study’s pulmonary function test results showed that normal pulmonary function tests (PFTs) were 201 (50.8%), restrictive lung diseases 100 (25.3%), obstructive lung diseases 78 (19.7%), and mixed were only 17 (4.3%), as detailed in Table 3.

**Table 1:** Demographic data of the patients.

	Frequency	Percent (%)
Age, years		
<20	36	9.1
20–39	146	36.9
40–59	116	29.3
≥60	98	24.7
Gender		
Male	208	52.5
Female	188	47.5
Total	396	100.0

**Table 2:** The distribution of the participants according to their body mass index (kg/m^2^) (n = 396).

Body mass index (kg/m^2^)	Frequency	Percent (%)
Underweight (<18.5)	96	24.2
Normal (18.5–24.9)	160	40.4
Overweight (25.0–29.9)	86	21.7
Obese (≥30)	54	13.6
Total	396	100.0

**Table 3:** Results of pulmonary function tests (n = 396).

Results of pulmonary function tests	Frequency	Percent (%)
Normal	201	50.8
Restrictive	100	25.3
Obstructive	78	19.7
Mixed	17	4.3
Total	396	100.0

**Figure 3.**
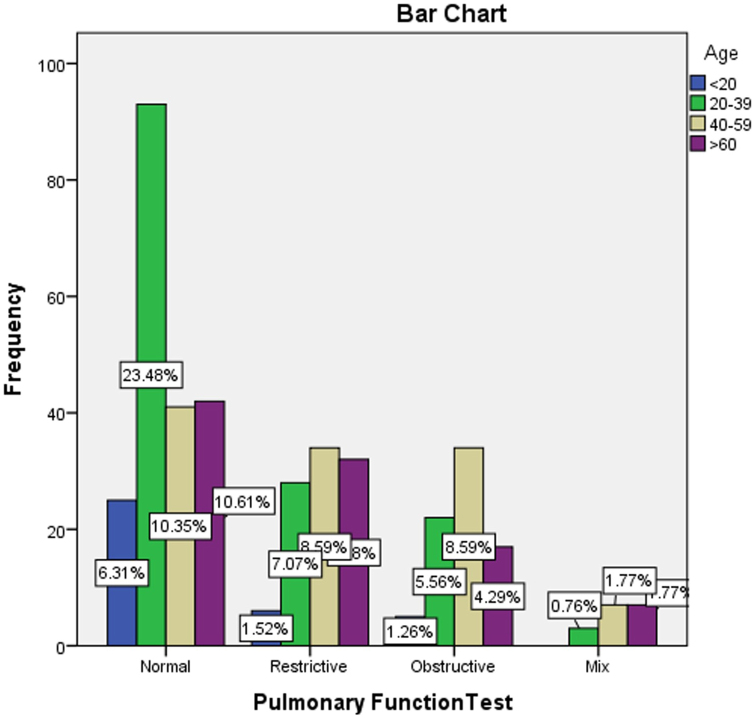
Age distribution in relation to pulmonary function test among participants.

**Figure 4.**
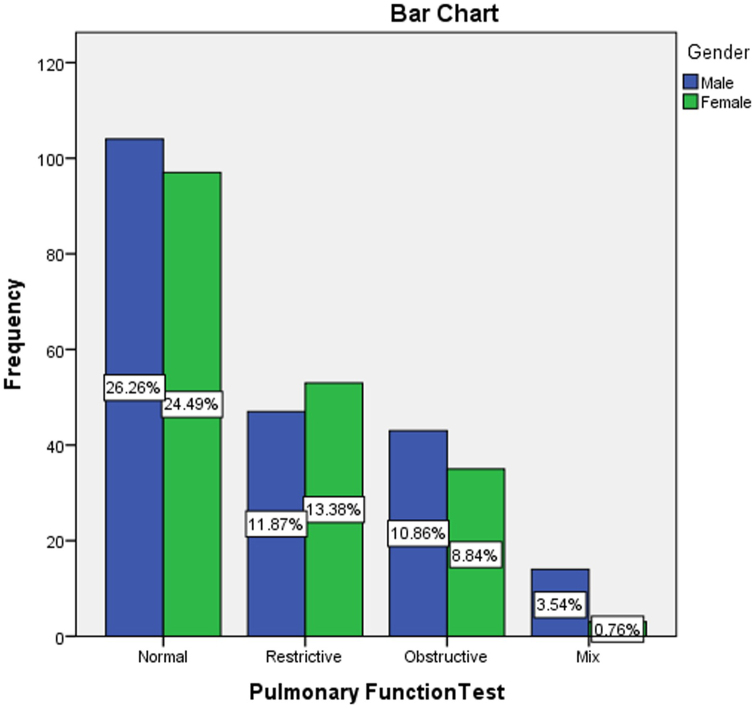
Gender distribution in relation to pulmonary function test among participants.

**Figure 5.**
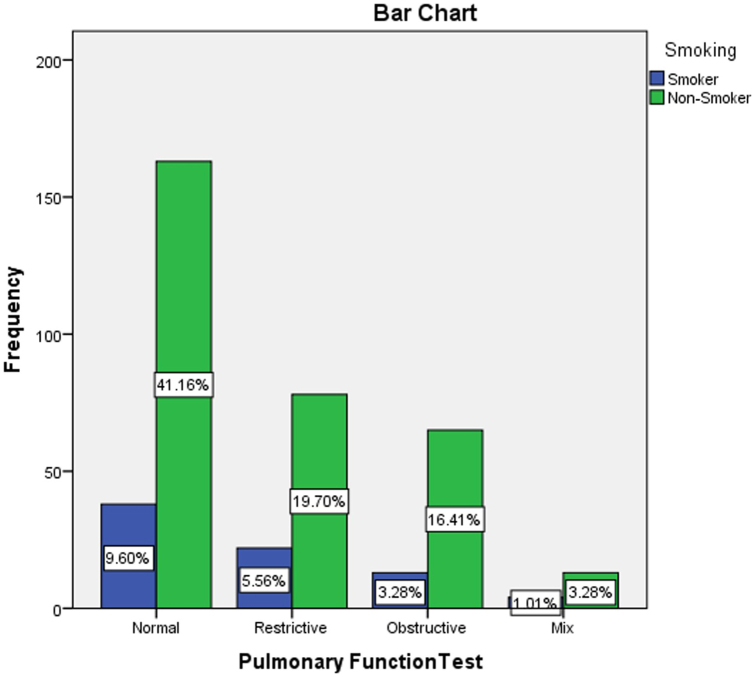
Smoking distribution in relation to pulmonary function test among participants.

**Figure 6.**
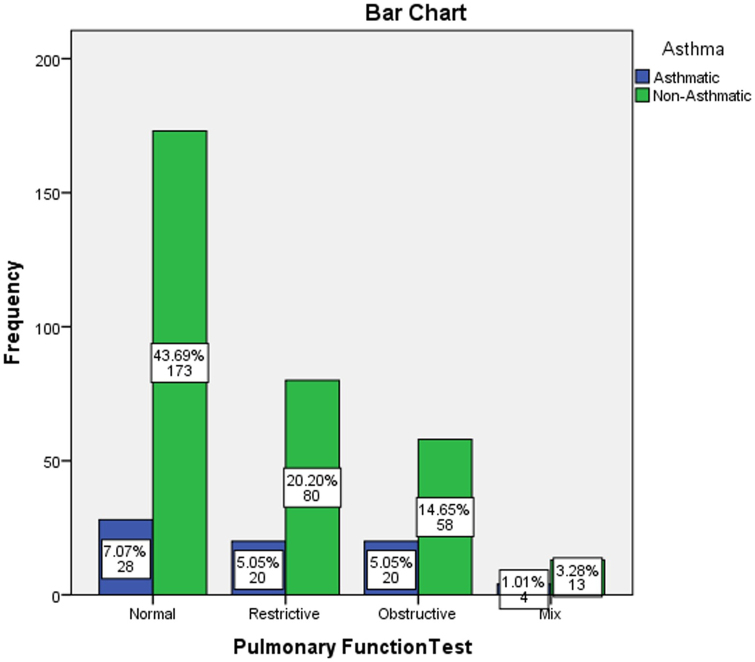
Asthma history distribution in relation to pulmonary function test among participants.

**Figure 7.**
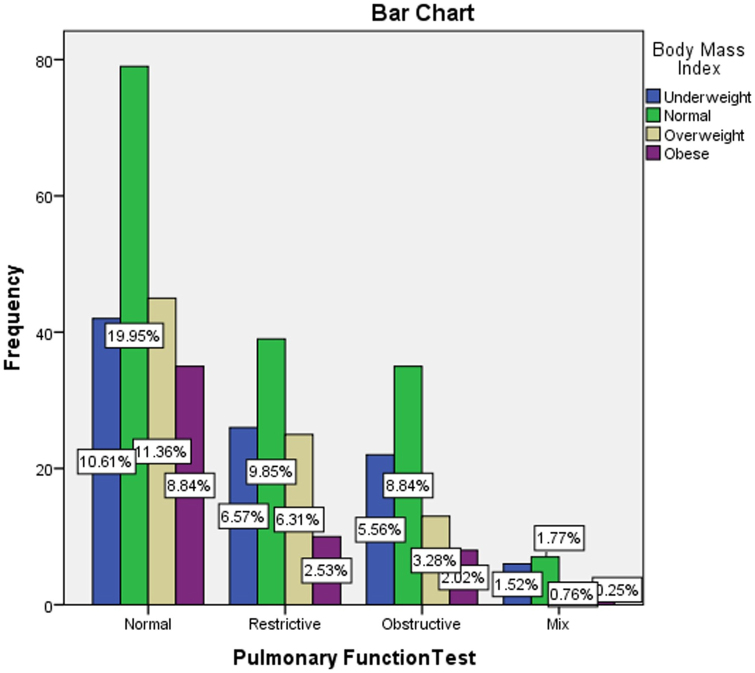
Body mass index distribution in relation to pulmonary function test among participants.

In this study, cross-tabulation was done to assess the possible association between the pulmonary function test results and some demographical and clinical characteristics of the participants using a chi-square statistical test. The analysis found a significant association between the older age of the participants and the abnormal pulmonary function test results (*P* < 0.001), as detailed in Table 4 and Fig. 3. The PFTs were similar across the participants’ genders, with a slight rise in male gender in mixed obstructive/restrictive PFTs results (*P* = 0.056), as shown in Table 5 and Fig. 4. Moreover, the study found that smoking was associated with some abnormal PFT results, such as restrictive/mixed, but the association was not significant (*P* = 0.797), as shown in Table 6 and Fig. 5. Also, a higher number of obstructive patterns was seen among patients with asthma; however, the association was not significant (*P* = 0.116), as in Table 7 and Fig. 6. Also, as in Table 8 and Fig. 7, abnormal PFTs were more common in low BMI patients, the association was, however, not significant (*P* = 0.440).

**Table 4:** The relation between the age of the participants with their results of the pulmonary functions tests (n = 396).

Age, years	Results of pulmonary function tests									
	Normal		Restrictive		Obstructive		Mix		Total	
	Freq.	%	Freq.	%	Freq.	%	Freq.	%	Freq.	%
<20	25	12.4	6	6.0	5	6.4	0	0.0	36	9.1
20–39	93	46.3	28	28.0	22	28.2	3	17.6	146	36.9
40–59	41	20.4	34	34.0	34	43.6	7	41.2	116	29.3
≥60	42	20.9	32	32.0	17	21.8	7	41.2	98	24.7
Total	201	100.0	100	100.0	78	100.0	17	100.0	396	100.0

Chi-square = 33.8387; *P* value < 0.001.

**Table 5:** The relation between the gender of the participants with their results of the pulmonary functions tests (n = 396).

Gender	Results of pulmonary function tests									
	Normal		Restrictive		Obstructive		Mix		Total	
	Freq.	%	Freq.	%	Freq.	%	Freq.	%	Freq.	%
Male	104	51.7	47	47.0	43	55.1	14	82.4	208	52.5
Female	97	48.3	53	53.0	35	44.9	3	17.6	188	47.5
Total	201	100.0	100	100.0	78	100.0	17	100.0	396	100.0

Chi-square = 7.5511; *P* value = 0.056.

**Table 6:** The relation between the smoking status of the participants with their results of the pulmonary function tests (n = 396).

Smoking	Results of pulmonary function tests									
	Normal		Restrictive		Obstructive		Mix		Total	
	Freq.	%	Freq.	%	Freq.	%	Freq.	%	Freq.	%
Smoker	38	18.9	22	22.0	13	16.7	4	23.5	77	19.4
Non-smoker	163	81.1	78	78.0	65	83.3	13	76.5	319	80.6
Total	201	100.0	100	100.0	78	100.0	17	100.0	396	100.0

Chi-square = 1.0196; *P* value = 0.797.

**Table 7:** The relation between the presence of asthma among the participants with their results of the pulmonary functions tests (n = 396).

History of asthma	Results of pulmonary function tests									
	Normal		Restrictive		Obstructive		Mix		Total	
	Freq.	%	Freq.	%	Freq.	%	Freq.	%	Freq.	%
Positive	28	13.9	20	20.0	20	25.6	4	23.5	72	18.2
Negative	173	86.1	80	80.0	58	74.4	13	76.5	324	81.8
Total	201	100.0	100	100.0	78	100.0	17	100.0	396	100.0

Chi-square = 5.9086; *P* value = 0.116.

**Table 8:** The relation between the body mass index of the participants with their results of the pulmonary functions tests (n = 396).

Body mass index	Results of pulmonary function tests									
	Normal		Restrictive		Obstructive		Mix		Total	
	Freq.	%	Freq.	%	Freq.	%	Freq.	%	Freq.	%
Underweight	42	20.9	26	26.0	22	28.2	6	35.3	96	24.2
Normal	79	39.3	39	39.0	35	44.9	7	41.2	160	40.4
Overweight	45	22.4	25	25.0	13	16.7	3	17.6	86	21.7
Obese	35	17.4	10	10.0	8	10.3	1	5.9	54	13.6
Total	201	100.0	100	100.0	78	100.0	17	100.0	396	100.0

Chi-square = 8.9736; *P* value = 0.440.

## Discussion

This study aimed to determine the prevalence of CRDs and the risk factors for these diseases and covered 396 participants.

In regard to the results of the pulmonary function test, our study showed that normal PFTs were 201 (50.8%), restrictive lung diseases 100 (25.3%), obstructive lung diseases 78 (19.7%), and mixed were only 17 (4.3%). Similarly, a study in Sudan and Tanzania by Egere *et al*. reported that unpublished data had shown an obstructive lung disease prevalence of 16.5 % in urban Sudan, and restrictive lung function was found in 55.6 % of adults who underwent spirometry^[9]^. Moreover, in Ethiopia, Getahun *et al*. reported that among participants, 24.3% had shown obstructive lung disease, and 22.9% had shown restrictive patterns in pulmonary functions^[10]^. Furthermore, lower rates were reported in SSA; Pefura *et al*. reported that the prevalence of restrictive patterns was 18.8%^[11]^. Also, in Nigeria, Ndukwu *et al*. reported that 37.9% of the participants had abnormal spirometry obstructive patterns in 40 (27.6%) and restrictive patterns in 10.3%^[12]^. Moreover, in Ghana, Antwi *et al*. found that one-quarter of the subjects (25.5%) had obstructive, 14.8% restrictive, and 11.7% exhibited combined forms of respiratory disease^[13]^. Also, this high frequency of obstruction is consistent with findings in Poland, where an obstructive spirometry pattern was found in 20.3% of participants^[14]^.

Our study found a significant association between the older age of the participants and the abnormal pulmonary function test results (*P* < 0.001). Likewise, in SSA, Pefura *et al*. reported that the determinants of the restrictive pattern included age ≥ 60 years^[11]^. Moreover, in Ghana, Antwi *et al*. found differences in the proportions of respiratory disorders for subjects in different age categories and patients who were 35 years and above, as well as restrictive and combined respiratory diseases^[13]^. In a similar context, in Brazil, Saulo *et al*. added that a cognitive deficit could have a negative effect on the quality of spirometry^[15]^. Similarly, Scarlata *et al*. found a prevalence of 10.9% for restrictive patterns among older people in the general population in Italy^[16]^. In the study by Mannino *et al*. comprising people aged 40 years and above from 14 sites across different continents who took part in the Burden of Obstructive Lung Disease study, the prevalence of restrictive pattern defined by a ratio forced expiratory volume in 1 second / forced vital capacity (FEV1/FVC) ≥ 70% and FVC < 80% varied from 6.1% in Sydney (Australia) to 29.3% in Cape Town (South Africa) and 45.9% in Manilla (Philippines)^[17]^. Soriano *et al*. also found a point prevalence of 12.7% in subjects aged 40 years and above in Spain^[18]^. The increasing prevalence of restrictive patterns with increasing age was very well characterized in the study by Mannino *et al*., where restrictive patterns were 2–2.5 times more frequent among the 50 and above compared with older participants^[19]^.

The study reported that the PFTs were similar across the participant’s gender, with a slight rise in male gender in mixed obstructive/restrictive PFTs results (*P* = 0.056). Likewise, in Ghana, Antwi *et al*. found that the respiratory diseases suggested in their study were not sex-dependent^[13]^. On the contrary, in Oman, Zakaria *et al*. reported that female patients exhibited lower mean values for all PFT parameters compared to male patients, and they concluded that males exhibited higher mean values of PFT parameters than females^[20]^. Moreover, the relationship between gender and abnormal spirometry has not been consistent. While some authors have found obstructive lung disease to be more common among females, we reported more males with obstructive patterns^[21]^. Although females are usually more exposed to biomass, some studies have reported a higher prevalence of obstructive lung disease in males during the period with a reversal at puberty that has been attributed to the effect of hormones^[22,23]^.

In our study, we found that smoking was associated with some abnormal PFTR results, such as restrictive/mixed, but the association was not significant (*P* = 0.797). In a similar context, in Turkey, Esra Dugral *et al*. added that smoking improves lung function in young adults; these are “healthy smokers.” Physical activity did not improve lung function, but the absence of physical activity significantly worsened lung function^[24]^.

Also, in our study, we report that the rate of asthma participants was higher among the obstructive PFTs results, but the association was not significant (*P* = 0.116). This high frequency of obstructive spirometry among these participants may also indicate that the prevalence of obstructive lung disease or asthma that has been reported among Nigerian people may have been underestimated^[25,26]^. Fabbri *et al*. agreed that typical examples of obstructive defects include COPD and asthma when a FEV1/FVC < 70% where FEV1 is reduced more than FVC signifies an obstructive defect^[27]^.

Also, in our study, we report that the participant’s BMI was slightly lower in participants with abnormal PFTs than those without significant dereference (*P* = 0.440). Likewise, in SSA, Pefura *et al*. reported that the determinants of restrictive patterns were underweight^[11]^. Moreover, in Ghana, Antwi *et al*. found differences in the proportions of respiratory disorders for subjects in different weight categories, whereas obstructive respiratory disease occurred more in obese patients^[13]^. Furthermore, in Saudi Arabia, Al Ghobain *et al*. concluded that obesity does not affect the spirometry tests (except peak expiratory flow) among healthy, non-smoking adults^[28]^. They recommend searching for alternative diagnoses in case of findings abnormal spirometry test results among obese subjects. On the contrary, in Oman, Zakaria *et al*. reported that increased BMI may be associated with a restrictive pattern on spirometry^[20]^. So, we show that there is a non-significant difference between underweight and overweight individuals who had both obstructive and restrictive conditions. Previous studies have also reported associations between body weight and various respiratory diseases. The weight of evidence suggests an association between obesity and obstructive lung disease^[29-31]^, except in very severe obesity (BMI > 40)^[32,33]^, where restrictive patterns emerge due to the impact of fat on respiratory function. On the other hand, underweight may predispose to restrictive or chronic obstructive forms of respiratory disease^[34-36]^.

The limitations faced by our study is that it was in a single center, which was purposively selected based on the heterogeneity of the population and centrality of its location in the state. The results may thus only be generalizable to some of the population in the other Sudan states. Along with that, the evaluation of smoking exposures and history of asthma symptoms are prone to recall bias and in providing information that could occur in many African settings.

## Conclusion

The analysis found a significant association between the older age of the participants and the abnormal pulmonary function test results. We concluded that the studies showed that the proportion of respiratory diseases with abnormal lung function test results is considerable and should not be ignored, especially among older patients. The relevant factors related to the abnormal results of the lung screening test, such as the elderly, should be considered to make them a priority in Sudan’s prevention and treatment policies. In addition to this, more extensive studies have been done to understand several other factors, such as the presence of various chronic diseases, occupational history, family history of respiratory diseases, history of using chronic treatments, and other various factors that directly or indirectly affect the results of lung function tests and prevalence rates of CLDs in Sudan. It is also better for the responsible authorities to provide the capabilities and equipment to perform lung functions routinely tests on a large scale in smaller hospitals, health centers, etc., to improve the ability for early detection of CLDs among different groups in society in Sudan.

## Data Availability

Available upon reasonable request.
